# Transcriptomic analysis reveals impact of gE/gI/TK deletions on host response to PRV infection

**DOI:** 10.1186/s12985-023-02265-y

**Published:** 2023-12-19

**Authors:** Xiaoli Wang, Yingguang Li, Shaoming Dong, Cong Wang, Yongming Wang, Hongliang Zhang

**Affiliations:** 1 Department of Preventive Veterinary Medicine, College of Veterinary Medicine, Shandong Agricultural University, Taian, China; 2 Shandong Collaborative Innovation Center for Development of Veterinary Pharmaceuticals, College of Veterinary Medicine, Qingdao Agricultural University, Qingdao, China; 3 China animal husbandry industry Co., Ltd, Beijing, China; 4 Shandong Huahong Biological Engineering Co., Ltd, Binzhou, China

**Keywords:** Pseudorabies virus, Mutant, gE/gI/TK, Gene deletions, Transcriptome, PK15 cells

## Abstract

**Background:**

Pseudorabies virus (PRV) causes substantial losses in the swine industry worldwide. Attenuated PRV strains with deletions of immunomodulatory genes glycoprotein E (gE), glycoprotein I (gI) and thymidine kinase (TK) are candidate vaccines. However, the effects of gE/gI/TK deletions on PRV-host interactions are not well understood.

**Methods:**

To characterize the impact of gE/gI/TK deletions on host cells, we analyzed and compared the transcriptomes of PK15 cells infected with wild-type PRV (SD2017), PRV with gE/gI/TK deletions (SD2017gE/gI/TK) using RNA-sequencing.

**Results:**

The attenuated SD2017gE/gI/TK strain showed increased expression of inflammatory cytokines and pathways related to immunity compared to wild-type PRV. Cell cycle regulation and metabolic pathways were also perturbed.

**Conclusions:**

Deletion of immunomodulatory genes altered PRV interactions with host cells and immune responses. This study provides insights into PRV vaccine design.

**Supplementary Information:**

The online version contains supplementary material available at 10.1186/s12985-023-02265-y.

## Introduction

Pseudorabies virus (PRV), also known as Suid herpesvirus 1 (SuHV1), belongs to the Herpesviridae family and is the causative agent of pseudorabies (PR) or Aujeszky’s disease (AD). Pigs are the natural host of PRV infection and the only animals that can survive PRV infection. PRV causes a highly contagious disease that severely threatens the pig industry, leading to reproductive failure, respiratory and neurological symptoms, and high mortality rates [1]. Infection of other animals with PRV results in acute, fatal disease with intense pruritus. Currently, attenuated live vaccines are the primary means of preventing and controlling pseudorabies [2].

The PRV genome can accommodate large foreign genes without compromising replicative ability, making it an ideal vector for expressing heterologous antigens. [3]. PRV vectors can be used to construct multivalent or broad-spectrum attenuated live vaccines to concurrently prevent PRV and infections by other important animal pathogens [4]. The PRV genome encodes 16 envelope glycoproteins that function in viral entry, egress and cell-to-cell spread. The gE glycoprotein is the major virulence protein enabling PRV to invade the host nervous system [5]. Deletion of the gE gene significantly decreases virulence and prevents invasion of the trigeminal and olfactory nerve terminals [6]. The gI-gE complex, together with gC, mediates viral release and impacts replication and virulence. Ablation of gI and gE functions dramatically affects PRV gene expression during infection. The gE glycoprotein can recruit the microtubule motor protein KIF1A to mediate retrograde axonal transport of PRV particles in neurons [6, 7]. gG is an immunomodulatory envelope protein that induces host cell secretion of interleukin-8 (IL-8) to attract neutrophil and monocyte migration and increase PRV infectivity [8]. gH is an envelope protein with fusion activity that can form a heterodimer with gL and, together with gB and gD, mediate PRV fusion with host cells [9]. gI is an envelope protein that facilitates cell-to-cell spread and retrograde neuronal spread, forming a heterodimer with gE and interacting with the gM/gN complex to impact intercellular PRV diffusion [10]. gK is an envelope protein that regulates viral budding and virulence, forming a heterodimer with UL20 and interacting with gB and gH/gL to impact intracellular transport and egress of PRV [11, 12]. gK can also affect PRV infection and virulence in the eyes, nose and throat [12].gL is an envelope protein with fusion activity that forms a heterodimer with gH and, together with gB and gD, mediates PRV fusion with host cells [9]. gM is an envelope protein that regulates viral budding and cell-to-cell spread, forming a heterodimer with gN and interacting with the gE/gI complex to impact intracellular transport and budding of PRV [13]. gN is an envelope protein that regulates viral budding and cell-to-cell spread, forming a heterodimer with gM and interacting with the gE/gI complex to impact intracellular transport and egress of PRV [13, 14]. The TK gene encodes a nonstructural protein with enzymatic activity to phosphorylate deoxynucleosides, participating in PRV DNA replication and transcription. TK impacts PRV latent infection and virulence [15]. TK-deleted or mutant PRV cannot establish latent infection in ganglia and exhibits attenuated virulence in mice and pigs [16]. TK utilizes host cell nucleotide metabolic pathways to provide necessary deoxyribonucleoside triphosphates (dNTPs) for PRV but can also convert certain antivirals like acyclovir (ACV) into active metabolites to inhibit PRV replication. Numerous PRV proteins are being continually explored for biological functions [17, 18]. Therefore,The glycoproteins gE and gI, along with the thymidine kinase (TK) gene, are major virulence determinants of PRV. gE enables neuroinvasion while TK impacts latent infection and virulence. Deletion of gE/gI/TK genes leads to dramatic attenuation of PRV.

In this study, we analyzed the transcriptomic changes in PK15 cells infected with the previously isolated virulent C strain, the attenuated SD2017gE/gI/TK strain with deletions of the major virulence determinants gE, gI and TK generated through homologous recombination using RNA-sequencing with Illumina platform. The goal was to elucidate the effects of key PRV virulence gene deletions on host cells, gain further insights into PRV pathogenesis, and establish a basis for novel attenuated live vaccine development.

## Materials and methods

### Virus and cell line

The highly virulent wild-type PRV mutant SD2017 strain was isolated in 2017 from the brains of PRV-infected piglets in Linyi, China [11]. SD2017gE/gI/TK was constructed in Shandong key laboratory of preventive veterinary medicine using homologous recombination to delete the gI, gE and TK genes, as described previously. PK15 (Sus scrofa epithelial kidney) cells used for PRV culture were obtained from the American Type Culture Collection (Manassas, VA, USA).

### Cell culture and virus infection

PK15 porcine kidney cells were cultured in high-glucose DMEM supplemented with 10% FBS and 1% penicillin-streptomycin. PRV SD2017 and PRV 2017gE/gI/TK were added at an MOI of 0.1 for 1 h, and the cells were then washed followed by the addition of 2% FBS / DMEM. PBS was used for mock infected control. Cells were harvested at 24 h post infection (hpi) in 3 independent biological replicates. RNA samples were extracted and stored at -80℃. the integrity, degradation and contamination of RNA were analyzed by agarose gel electrophoresis. The purity of RNA (OD260/280 and OD260/230 ratio) was detected by NanoDrop ND-1000 spectrophotometer (Nano Drop Inc., Wilmington, DE, USA). Agilent 2100 Bioanalyzer system (Agilent Technologies, Santa Clara, CA, USA) was used to accurately detect 28 S/18 or 23 S/16S and RIN values, and accurately detect RNA integrity.

### Library construction and transcriptome sequencing

A total amount of 1 µg RNA per sample was used as input material for the RNA sample preparations. Sequencing libraries were generated using NEBNext® UltraTM RNA Library Prep Kit for Illumina® (NEB, USA) following manufacturer’s recommendations, and index codes were added to attribute sequences to each sample [19].This kit was used to prepare sequencing libraries from total RNA. In order to select cDNA fragments preferably 250 ~ 300 bp in length, the library fragments were purified with AMPure XP system (Beckman Coulter, Beverly, USA). Considering shorter fragments contain less sequencing information, we optimized conditions to obtain ideal 300–400 bp fragments, which allows richer sequencing information while ensuring quality. Then 3 µl USER Enzyme (NEB, USA) was used with size-selected, adaptor-ligated cDNA at 37 °C for 15 min followed by 5 min at 95 °F before PCR. Then PCR was performed with Phusion High-Fidelity DNA polymerase, Universal PCR primers and Index (X) Primer. Finally, PCR products were purified (AMPure XP system), and library quality was assessed on the Agilent Bioanalyzer 2100 system. We ensured all sample libraries met requirements for subsequent sequencing [20, 21].

The index-coded libraries were pooled and clustering was performed on a cBot Cluster Generation System using TruSeq PE Cluster Kit v3-cBot-HS (Illumina). After cluster generation, the library preparations were sequenced on an Illumina Novaseq platform generating 150 bp paired-end reads. Downstream quality control and information analysis were carried out to ensure accuracy of analysis. [21, 22].

### RT-qPCR validation of differentially transcribed genes

According to the sequencing results, nine genes with increased or decreased transcription levels were randomly selected for validation of the high-throughput sequencing data. Glyceraldehyde-3-phosphate dehydrogenase (GAPDH) was used as the internal reference gene. Primers were designed using Premier 6.0 software (Table 1). After RNA extraction and quality control, total RNA samples were reverse transcribed into cDNA using the HiScript® II Q RT SuperMix kit (Vazyme, Nanjing, China) following the manufacturer’s protocol. The obtained cDNA samples were aliquoted and stored at -80 °C for subsequent qRT-PCR validation.The cDNA was used as template for quantitative real-time PCR (qPCR) assays performed with the SYBR Green PCR kit (Vazyme) on the QuantStudio 5 Real-Time PCR System (Thermo Fisher Scientific). The relative transcription levels of each gene were calculated by the 2-^ΔΔCt^ method. Statistical analysis was conducted using GraphPad Prism version 5.0 to compare transcription levels between groups by t-test.

**Table 1:** Primers for real-time PCR

Gene name	Primer	Primer sequence (5′→ 3′)	source
CCL20	F	AGCAACTTTGACTGCTGCC	PMID: 29,997,306
	R	GATCTGCACACACGGCTAA	
ISG15	F	GGTGCAAAGCTTCAGAGACC	PMID: 32,575,635
	R	CCTCGAAAGTCAGCCAGAAC	
CD80	F	AGCGGGAGAGAGGGTCTTAT	PMID: 22,925,563
	R	AAGGGCAGTAATACTAGGCAC	
STAT1	F	AAATCAGGACTGGGAGCACG	Self-Designed
	R	CTTGCTTTTCCTAATGTTATGCT	
IL6	F	TGGCAGAAAAAGACGGATGC	PMID: 29,997,306
	R	ACAGCCTCGACATTTCCCTT	
FGFR2	F	GGTCCATCAATCACACATACCACC	PMID: 33,107,129
	R	TGGGGCTGGGCATCACTGTA	
CD40	F	TCAAGCAGATGGCGACAGAG	PMID: 22,925,563
	R	CACCAGGGCTCTCATCCGA	
PRV-gC	F	CTCTTCGGTGAGCCCTTCC	Self-Designed
	R	GTGCTGTTGGTCACGAAGGC	
GAPDH	F	CCCCTTCATTGACCTCCACTA	Self-Designed
	R	CATTTGATGTTGGCGGGAT	

### Data analysis

The unprocessed fastq raw data were fed into homemade Perl scripts for preprocessing. We retrieved the Sus scrofa 11.1 reference genome and annotations from the Ensembl database. Hisat2 was utilized to align the cleaned reads against the reference genome. FeatureCounts software was leveraged to tally the number of reads mapping to each gene. The FPKM metric for each gene was obtained by factoring in length and aligned reads. We carried out differential expression analysis between conditions by means of the DESeq2 R package. DESeq2 identifies differentially expressed genes from count data by wielding a negative binomial model. P-values were modified using the Benjamini-Hochberg procedure to rein in the false discovery rate. Genes with adjusted p-values below 0.05 were tagged as differentially expressed. Enrichment analysis of the DEGs for GO terms was executed with clusterProfiler, rectifying for gene length bias. Significantly enriched GO terms exhibited adjusted p-values < 0.05. The KEGG resource was tapped into for elucidating high-level functionalities of biological systems. clusterProfiler was utilized to evaluate KEGG pathway enrichment among the DEGs.

## Results

### Sample infectivity performance evaluation

**Fig. 1:**
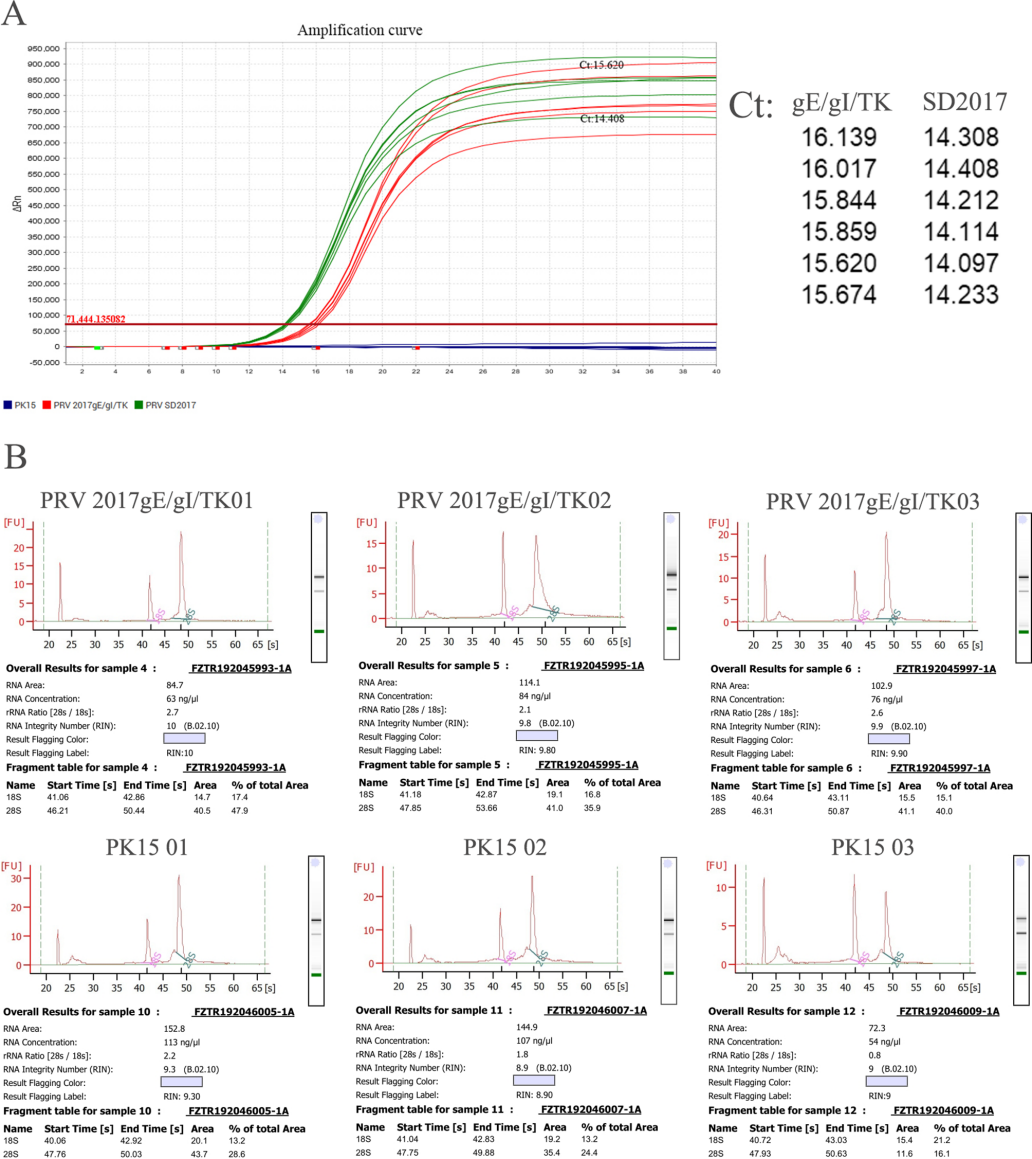
Sample assessment and RNA integrity analysis of the samples. (**A**) The deletion of PRV’s gE/gI/TK genes affected the viral copy number in cells under identical infection states. Biological replicates were performed in triplicate and technical replicates in duplicate; no viral nucleic acids were detected in the PK15 control group. (**B**) Each subplot shows the RNA electrophoresis graph and RIN value of a sample. The title of the subplot is the sample number, such as “PRV 2017 gEA/gI/TK01”. In the graph, the pink and green peaks represent 28 S and 18 S rRNA, respectively. The linear regression curve is used to calculate the RIN value, which indicates the level of RNA integrity. The higher the RIN value, the better the RNA quality. Generally speaking, RNA with a RIN value greater than 7 can be used for subsequent experiments

qPCR was used to assess the intracellular PRV replication levels in samples infected for the same duration by both the PRV 2017gE/gI/TK and PRV SD2017 strains. The original virus strain exhibited significantly higher replication levels compared to the virulence gene-deleted strain, with its highest CT value at 14.408 and the lowest CT value for the deletion strain at 15.620 (Fig. 1A).

### Quality control of sequencing data

RNA was extracted from PK15 cells infected with PRV 2017gE/gI/TK and control groups. The RNA integrity was assessed by measuring the ratio of 28 S to 18 S rRNA and the RIN value. As shown in Fig. 1B, the ratio of 28 S to 18 S was greater than 1.5 for all samples except PK15 03, which had a ratio of 0.8. However, the RIN value of PK15 03 was 9, indicating high RNA quality. The other samples also had RIN values above, which is generally considered acceptable for downstream experiments. The data has been submitted to the SRA database, with the accession number PRJNA1001590.

### Differential expression analysis

**Fig. 2:**
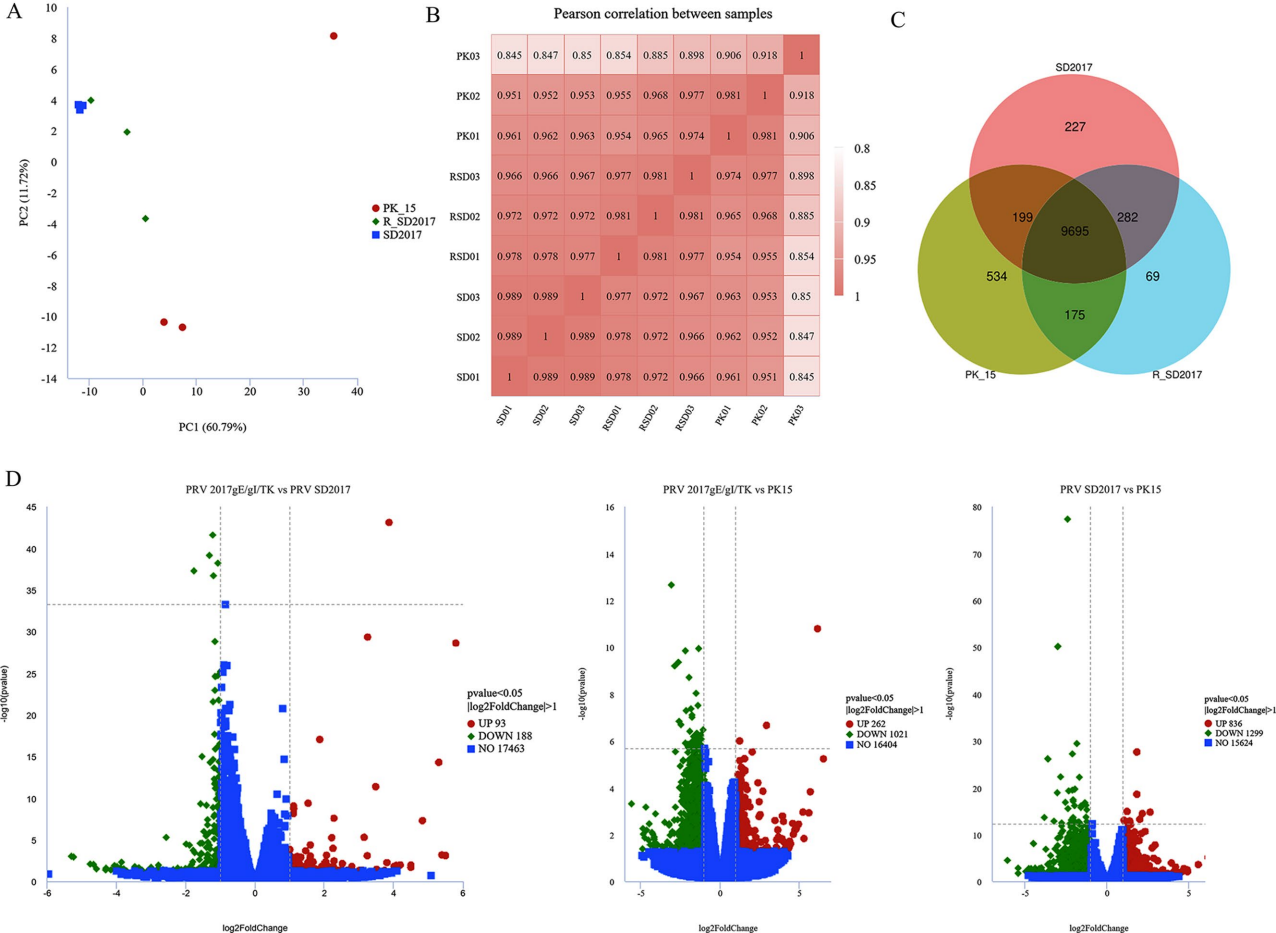
Differential expression analysis of samples. (**A**) Sample dispersion analysis. (**B**) Heatmap of sample differential expression analysis. (**C**) Venn diagram of DEGs between the 3 sample groups. (**D**) DEGs screening for PRV 2017gE/gI/TK vs. PRV SD2017, PRV 2017gE/gI/TK vs. PK15 and PRV SD2017 vs. PK15. Criteria were set as |log2 fold change| > 1 and adjusted p-value < 0.05

To screen for differentially expressed genes (DEGs) in PK15 cells after infection with PRV 2017gE/gI/TK And PRV SD2017 we used the DESeq2 software package to perform differential expression analysis of the transcriptome se-quencing data and plotted the results. Principal component analysis showed the PRV 2017gE/gI/TK deletion mu-tant, wild-type PRV, and PK15 groups were closely clustered (Figure 2A). Therefore, this study focused on the dif-ferences between the attenuated PRV 2017gE/gI/TK strain and the wild-type PRV SD2017 strain, as well as the PK15 control group (Figure 2B). Venn diagrams displayed the DEGs between the 3 groups (Figure 2C). We set the screen-ing criteria for differential expression as |log2 fold change | > 1 and adjusted p-value < 0.005. According to these criteria, we screened the PRV 2017gE/gI/TK vs PRV SD2017 comparison and identified 93 up-regulated and 188 downregulated genes, the PRV 2017gE/gI/TK vs PK15 comparison showed 262 upregulated and 1021 downregulated genes, and the PRV SD2017 vs PK15 comparison showed 836 upregulated and 1299 downreg-ulated genes. Volcano plots were used to show the number and distribution of DEGs between groups (Figure 2D).

### GO analysis of DEGs

**Fig. 3:**
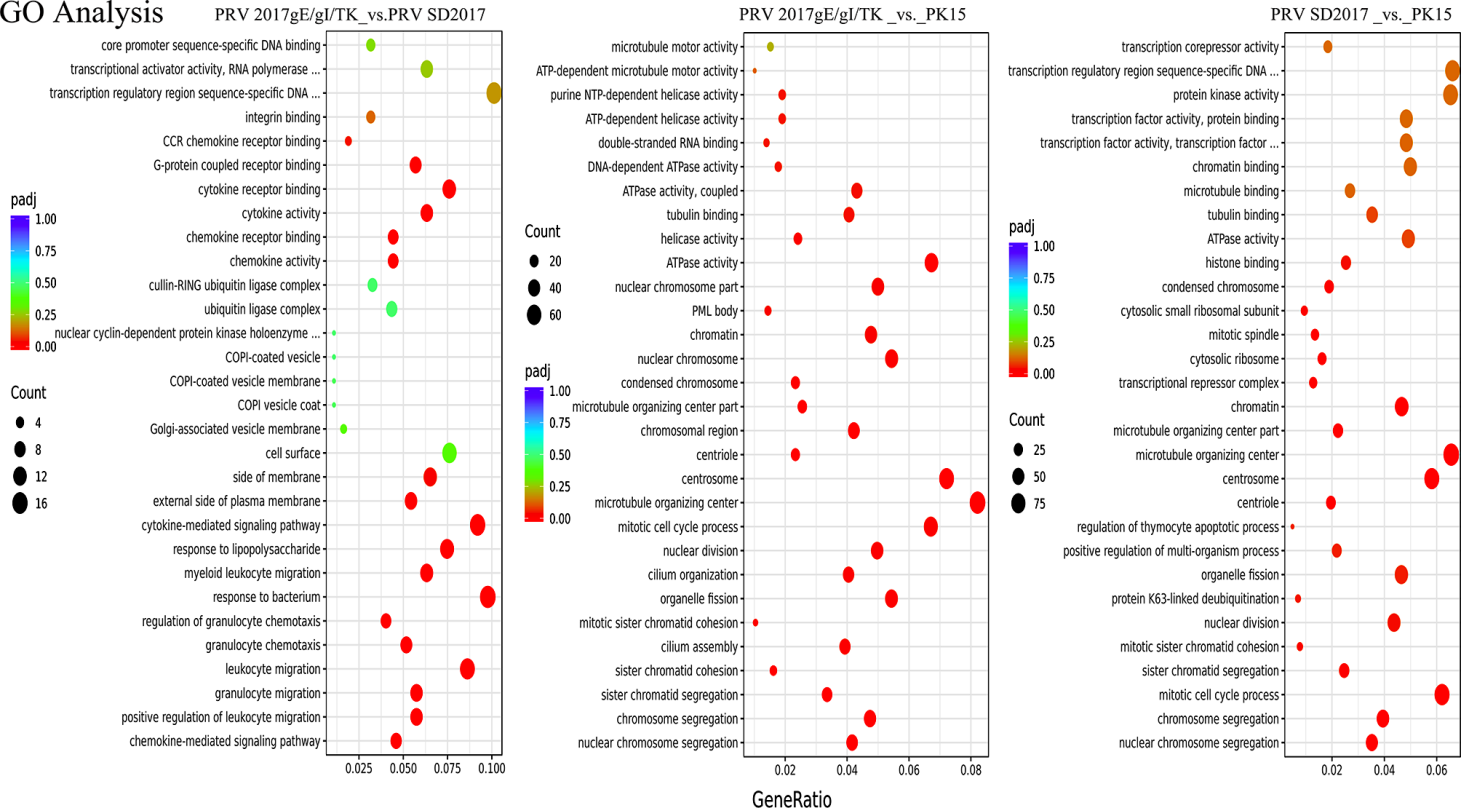
Top 30 GO analysis. GO enrichment analysis comparing PRV 2017gE/gI/TK vs. PRV SD2017, PRV 2017gE/gI/TK vs. PK15 and PRV SD2017 vs. PK15. The x-axis shows the GO terms. The y-axis shows the significance level of GO term enrichment. Higher values indicate more significant enrichment. and the color from red to purple represents the significance of the enrichment. Criteria were set as |log2 fold change| > 1 and adjusted p-value < 0.05

The GO analysis mainly focused on functional annotation differences between the attenuated PRV 2017gE/gI/TK strain and the virulent PRV SD2017 strain, primarily by comparing the top 30 significant differences in PRV 2017gE/gI/TK vs. PRV SD2017 and PRV 2017gE/gI/TK vs. PK15, and PRV SD2017 vs. PK15 (Fig. 3). The GO analysis revealed that both infections significantly enriched processes highly relevant to DNA replication and damage repair, like cell cycle checkpoint (GO:0000075) and DNA repair (GO:0006281). These results indicate the viral infection jeopardized the genome integrity of host cells. Additionally, altered RNA splicing and processing (GO:0008380) and cytoskeleton organization and dynamics (GO:0000226) were observed, suggesting the viruses likely hijacked host RNA processing and intracellular trafficking systems. Some immune and inflammatory processes (GO:0006955) were also enriched, especially in the virulent strain infection, reflecting the immune responses elicited by the viruses. Moreover, modulated signal transduction pathways (GO:0007173) and protein degradation pathways (GO:0030163) manifested the extensive effects of viral infections on host cells. In summary, the GO analysis portrayed how the virulent strain intricately manipulated host immunity, genome stability, signal transduction, etc., inflicting more severe infection and damage to host cells. The loss of virulence genes in the attenuated strain may contribute to these observations.

### KEGG analysis of DEGs

**Fig. 4:**
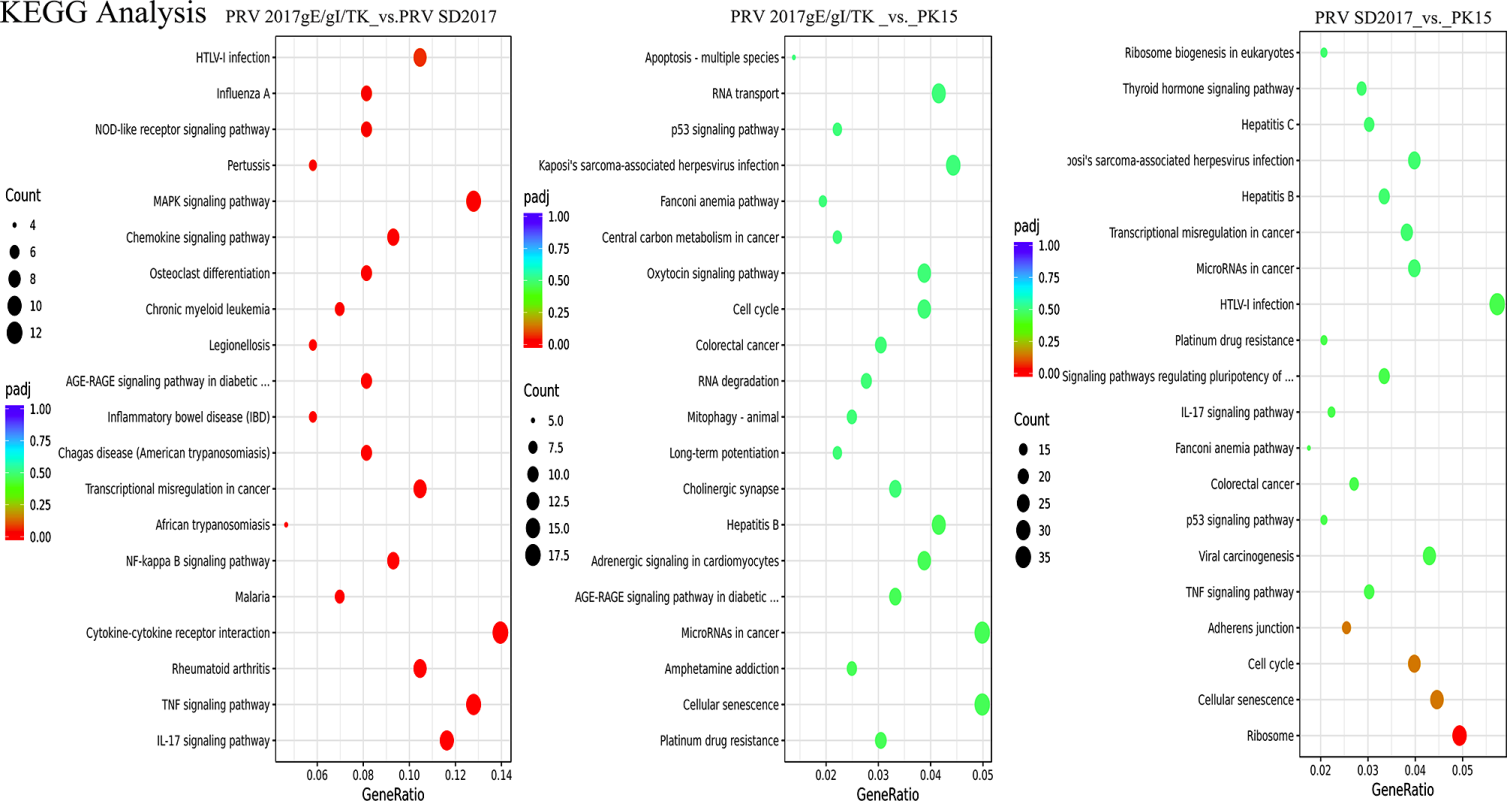
Top 20 KEGG analysis. KEGG enrichment analysis comparing PRV 2017gE/gI/TK vs. PRV SD2017, PRV 2017gE/gI/TK vs. PK15 and PRV SD2017 vs. PK15. The x-axis shows the ratio of differentially expressed genes annotated to each KEGG pathway versus total differentially expressed genes. The y-axis shows the KEGG pathways. Dot size represents the number of genes annotated to each pathway. Dot color from red to purple indicates increasing enrichment significance. Criteria were set as |log2 fold change| > 1 and adjusted p-value < 0.05.3.6. Interaction analysis of differentially expressed proteins

KEGG analysis mainly focused on the pathway annotation differences between PRV 2017gE/gI/TK weak strain and PRV SD2017 by analyzing the significant differences in top 20 pathways between PRV 2017gE/gI/TK vs. PRV SD2017 and PRV 2017gE/gI/TK vs. PK15, PRV SD2017 vs. PK15 as shown in (Fig. 4). For virulent strain vs. blank, cell cycle pathway was significantly enriched, with genes GADD45B, CDC7, CCNB3. Focal adhesion pathway was also significantly enriched, with genes SNAI1, TGFBR1, CTNND1. Serine protease inhibitor aging pathway was significantly enriched with gene GADD45B. For attenuated strain vs. virulent strain, IL-17 signaling pathway was significantly enriched, with inflammatory genes CCL20, CXCL2, CXCL8 significantly upregulated. TNF signaling pathway was significantly enriched, with inflammatory genes CCL20, CXCL2, TNF significantly upregulated. Chemokine signaling pathway was significantly enriched, with inflammatory genes CCL20, CXCL8, CXCL10 significantly upregulated. Enriched pathways also included rheumatoid arthritis, pathogen recognition receptor pathways and other immune-related pathways. For attenuated strain vs. blank PK15 cells, significantly enriched pathways included: Drug resistance pathway (ko01524), with genes MSH2, BIRC3 upregulated; Cellular senescence (ko04218), with genes SIRT1, NBN upregulated; cAMP signaling pathway (ko04024), with genes FOS, PDE4D upregulated. Analysis of virulent strain vs. blank showed virulent strain disrupted host basic survival functions, which may be due to higher replication efficiency and more damage to host cells. The enrichment of immune and inflammatory pathways and significant upregulation of inflammatory genes like CCL20, CXCL2, CXCL8 in attenuated strain can serve as evidence for easier recognition and clearance of attenuated strain by host (Fig. 4).

**Fig. 5:**
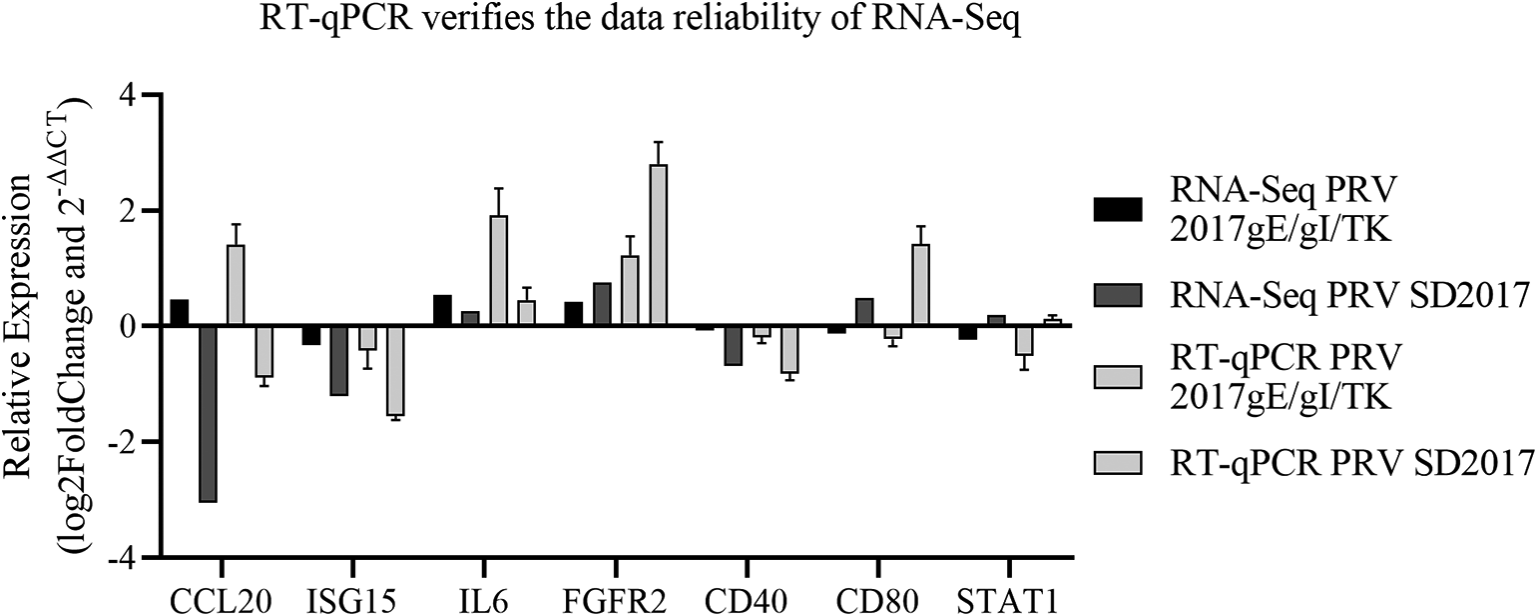
Comparison of DEGs fold changes between RNA-Seq and RT-qPCR. PK-15 cells were infected with PRV SD2017 and PRV 2017gE/gI/TK for 24 h, then RT-qPCR was performed to detect relative expression of selected DEGs. RT-qPCR data are from three independent experiments, with GAPDH as control. Differences were evaluated by ANOVA. The horizontal axis shows the DEG names. The vertical axis indicates log2-fold changes of DEGs and 2-^ΔΔC^t values

### Validation of the expression of DEGs by RT-qPCR

To further validate the transcriptome analysis results, we performed a RT-qPCR analysis to determine the reproducibility of the differential gene expression. GAPDH mRNA was amplified as the endogenous control. Four down-regulated genes (STAT1, CD80, CD40, FGFR2) and three up-regulated genes (IL6, ISG15, CCL20) identified in RNA-seq were selected for RT-qPCR verification. The RT-qPCR results showed that the expression trends of these 7 genes were consistent with the RNA-seq data, though the extent of up/down-regulation varied. Therefore, the RNA-seq data was considered reliable for screening the differentially expressed genes. This RT-qPCR verification demonstrated the reproducibility and accuracy of our transcriptome analysis results.

## Discussion

Pseudorabies virus (PRV) is a pathogen that causes up to 50% mortality in newborn piglets, severely impacting the pig industry. The Bartha-K61 vaccine protects against lethal infection by classical PRV strains but not emerging variants [19]. Gene knockouts of gE/gl/TK successfully attenuate PRV, and the TK gene is an important virulence factor in PRV variants. In PRV-sensitive mice, TK knockout mutants completely lost pathogenicity [20, 21]. Vaccines lacking gE showed significantly reduced infection of second- and third-order neurons in the olfactory and trigeminal pathways [21, 22]. For protection of suckling piglets, a live vaccine with gl/gE/TK mutations provided more complete protection from lethal challenge by variants than the Bartha-K61 vaccine [23]. PRV lacking key virulence genes gE, gI and TK exhibited attenuated growth kinetics in PK15 cells [24, 25]. In previous studies, we also evaluated the immunoprotective efficacy of the PRV SD2017ΔgE/gl/TK attenuated live vaccine candidate, further confirming deletion of gE/gl and TK as a suitable scheme for PRV vaccine development [26].

The development of transcriptomics enables us to explore the impact of gE/gl/TK gene knockouts on the PRV infection process and makes us more interested in comparing attenuated strains with wild-type strains [11] In this study, functional enrichment analysis of PRV SD2017gE/gl/TK and PEDV SD2017 by transcriptomics showed enrichment of inflammatory response and response to bacteria in the PRV SD2017 infection group, indicating the wild-type strain can more effectively stimulate host inflammatory response. Viral life cycle was also enriched, suggesting enhanced viral life cycle process during infection with the virulent strain. Compared to wild-type, the attenuated PRV 2017gE/gI/TK strain showed significant enrichment in IL-17, TNF and chemokine signaling pathways, with upregulation of inflammation-related genes CCL20, CXCL2, CXCL8. The increased expression of cytokines like CCL20, CXCL2 and chemokine pathways suggests that deletion of immunomodulatory gE/gI/TK genes facilitates immune recognition of attenuated PRV strains. This is consistent with the known roles of gE/gI/TK in immune evasion and indicates defects in virulence upon gene deletion. This finding is consistent with past research showing PRV can induce macrophage responses that may participate in inducing inflammatory adaptive host defenses [27].Further KEGG enrichment analysis comparing the attenuated strain to PK15 and PRV SD2017 to PK15 showed the attenuated strain enriched pathways related to tumors, cell cycle, and cell senescence compared to PK15. These included tumor pathways (colorectal cancer, pancreatic cancer, etc.) and p53 signaling, cell cycle, AMPK signaling pathways, reflecting the attenuated strain infection has some impact on host cell proliferation and metabolic regulation. Compared to the virulent strain, the attenuated strain enriched more immune and inflammatory pathways, including IL-17 signaling, TNF signaling, chemokine signaling, Toll-like receptor signaling, etc., indicating stronger activation of host immune responses by the attenuated strain. The enhanced immune pathways in our analysis may be mainly due to differences in immunostimulatory effects caused by the lack of virulence genes in the attenuated strain. Previous studies showed that activation of cytosolic DNA sensing and Nod-like receptor signaling may be involved in PRV recognition, while NF-kB and TNF signaling activation may participate in antiviral immune responses [28]. PRV has been shown to inhibit NFkb replication activity by ubiquitinating cGAS [20]. HSP27 as a key PRV gene can weaken cGAS-mediated IFN-β signaling by ubiquitinating cGAS, promoting PRV infection [29]. The RNA helicase DDX56 inhibits PRV replication by regulating IFN-β signaling through targeting cGAS [30]. Cholesterol 25-hydroxylase acts as a host restriction factor inhibiting PRV replication [31]. Porcine IFITM1 inhibits PRV infection as a host restriction factorwhile TMEM41B promotes PRV replication as an interferon-stimulated gene [32, 33]. ENPP1 maintains cGMP-AMP homeostasis involved in PRV infection [34]. Though discovered in 1902 and considered an “old virus”, our knowledge of pseudorabies virus (PRV) remains limited regarding its interactions with host cells, protein functions, and strategies for evading host immunity as a veteran pathogen [20]. In recent years, with the emergence of variant strains causing outbreaks, research has focused more on immunoprotection conferred by gene knockouts of virulence factors, and development of novel vaccines, while less attention has been paid to changes in virus-host interactions after deletion of virulence genes. In this study, we revealed modulation of host cell transcriptional regulators from omics data, aiming to gain in-sights into PRV pathogenesis mechanisms and provide a rationale for developing novel live attenuated PRV vaccines.

## Conclusions

Transcriptomic DEGs analysis of PK15 cells infected with PRV having gE/gI/TK gene deletions versus wild-type PRV SD2017 indicates the gE/gI/TK deletions result in defects in the invasive and immune evasion abilities of PRV, making it more likely to stimulate host immune responses and impact host cell cycle and metabolic pathways. PEDV SD2020 appears able to more strongly activate inflammatory and immune responses. This study promotes exploration of the functions of the gE/gI/TK deletion proteins and establishes a foundation for investigating PRV pathogenesis mechanisms.

### Electronic supplementary material

Below is the link to the electronic supplementary material.

**Supplementary Material 1:** Group Comments Supplementary

**Supplementary Material 2:** RSD2017vsPK15_all_GOenrich Supplementary

**Supplementary Material 3:** RSD2017vsPK15_all_KEGGenrich Supplementary

**Supplementary Material 4:** RSD2017vsSD2017_all_GOenrich Supplementary

**Supplementary Material 5:** RSD2017vsSD2017_all_KEGGenrich Supplementary

**Supplementary Material 6:** SD2017vsPK15_all_GOenrich Supplementary

**Supplementary Material 7:** SD2017vsPK15_all_KEGGenrich

## Data Availability

The transcriptome data can be accessed from the SRA database using the accession number PRJNA1001590.
